# How has the COVID-19 pandemic improved evidence-based-medicine awareness among undergraduate medical students?

**DOI:** 10.1080/10872981.2020.1787123

**Published:** 2020-06-29

**Authors:** Maryam Fourtassi, Ghita Hjiej, Youness Touissi, Abderrazak Hajjioui, Naima Abda

**Affiliations:** a Faculty of Medicine and Pharmacy of Oujda, Mohammed Premier University, Oujda, Morocco; b Laboratory of Epidemiology, Clinical Research and Public Health, Mohammed Premier University, Oujda, Morocco; c Faculty of Medicine and Pharmacy of Rabat, Mohammed V University, Rabat, Morocco; d Laboratory of Clinical Neurosciences, Faculty of Medicine and Pharmacy of Fez, Sidi Mohamed Ben Abdellah University, Fez, Morocco

**Keywords:** COVID-19, evidence-based medicine, undergraduate medical students

## Letter to the editor

Evidence-based medicine (EBM) is a key component of current medical practice, as it induces effective decision-making regarding patients care, based on the best available evidence [1]. However, delivering EBM in the undergraduate curriculum is a real challenge, especially in settings where undergraduate medical education is mainly based on textbooks-like information that is usually presented to students as solid and verified knowledge with less room to doubt and uncertainty. Medical information is usually taught to undergraduates as a set of general truths they can build their analytic thinking around in order to reach a diagnosis or an intervention decision. Hence, the transition from theory to ‘bedside’ practice becomes difficult and might cause a real shock to some learners when they see that a patient could actually have the condition without it fitting the exact textbook description, or when they discover that an antibiotic is not always 100% efficient as they might have thought.

While early introducing undergraduate medical students to the basics of EBM is important to improve their critical thinking [2], and raise their awareness about how medical knowledge is actually built over years of translational research, it might seem difficult to make them understand and accept its common frustrating pitfalls, and to explain how these limitations are part of the whole process effectiveness. Interestingly, this current ongoing COVID-19 pandemic represents a comprehensive and easily perceptible teaching example.

Despite its worldwide devastating health and socio-economical consequences, the COVID-19 pandemic has brought a great opportunity to promote the vital importance of EBM among undergraduate medical students, who have found themselves in the middle of an international research race to face the virus. Each of the phases this global health crisis has crossed comprises a valuable basic EBM lesson we can teach our undergraduate students, with higher chances for them to understand and integrate it, even if they have not been used to it before. Moreover, this pandemic has brought to students’ attention the vital need of often overlooked disciplines in their learning such as ‘epidemiology’, ‘biostatistics’, ‘critical reading’ or even ‘medical English’ for those studying in non-English-speaking countries like Morocco [3], to be able to understand scientific resources.

Thanks to the COVID-19 pandemic, our undergraduate students understand better how mastering the exact modes of the virus transmission can impact the choice of prevention measures and how strictly they should be implemented to contain the viral contamination spread. They are also aware of how critical physiopathology knowledge is to defining the best treatment approaches, and choosing the most effective drugs to prescribe. They better understand how symptoms can be misleading, and how atypical clinical presentations should always be considered especially when the patient’s life is endangered.

On the other hand, the global multi-centred clinical trials to identify the best therapy against the disease and the basic-science-laboratories race to find a vaccine are even richer of key messages regarding the inseparable vulnerability and strength of fundamental and clinical research. Indeed, the scientific evidence provided by research results can be affected by some limitations, mainly related to the lack of follow up and insufficient observation time and materials regarding a recent and unknown phenomenon, which makes decisions and recommendations evolve and change with every new piece of information that can be a source of new evidence. Hence, this commonly used sentence: ‘more research is needed to conclusively establish … ’ that is usually added in every research paper conclusion has never been as meaningful as today with this unlimited Coronavirus-related research papers production, providing complementary and sometimes paradoxical information to be carefully examined and interpreted.

Today, our students are aware that published evidence still needs to be confirmed or infirmed by further investigations, and that publishing in a high impact journal does not make your results or conclusions beyond scientific criticism, leading to possible paper retraction [4,5]. They are also aware of the multiple levels of evidence every kind of research can provide, and that research can be biased by conflicts of interests that should be controlled and fully declared. Today, our undergraduate students can see that despite its limitations, the scientific research and publishing system is still strong by implementing a double security process, with the prior publishing peer-review and post publishing possibility for the scientific community to openly discuss and criticise peer-productions through scientific correspondence. These security and quality guarantors are the main source of research strength and a good reason to convince our students of its importance.

One could argue that those key lessons about research and EBM can still be depicted by other older pandemic examples and yet, only when you are personally involved in the experience, that you really get it deeply integrated. This pandemic experience has thought to medical students over the world about EBM and research more than they could ever learn through their Medical Schools’ theoretical settings. And we should all take this opportunity to draw lessons and teachings that we can share with our students.
